# Ghostwriting Revisited: New Perspectives but Few Solutions in Sight

**DOI:** 10.1371/journal.pmed.1001084

**Published:** 2011-08-30

**Authors:** The PLoS Medicine Editors

## Abstract

The *PLoS Medicine* editors discuss new perspectives on ghost writing and reflect on the suggested remedies put forth in this month's issue of the journal.

Two years ago, in an editorial [1] sparked by the revelations from *PLoS Medicine* and *The New York Times*' intervention in litigation relating to Prempro [2], we wrote that “the story told in these documents amounts to one of the most compelling expositions ever seen of the systematic manipulation and abuse of scholarly publishing by the pharmaceutical industry and its commercial partners in their attempt to influence the health care decisions of physicians and the general public.” In the first scholarly examination of these documents, published late last year [3], Adriane Fugh-Berman concluded that “marketing messages in credible journals have almost certainly contributed to widespread use of hormone replacement therapy among millions of women who had no medical indication for the drug” – a statement that suggests the medical literature had been acting in direct contradiction of the first ethical rule of all physicians, to “first, do no harm.”

Over the past month *PLoS Medicine* has published three articles that bring new perspectives to the problem of ghostwriting. These perspectives and the possible remedies that two of them offer need serious consideration in light of two recent conferences [4],[5] and other evidence suggesting that, in stark contrast to the protestations of many in the pharmaceutical and medical writing industries, ghostwriting and its larger relation, ghost-management, of the medical literature remain key tactics deployed by pharmaceutical companies, and that current attempts to reduce the practices are not succeeding.

The first article, by Simon Stern and Trudo Lemmens [6], takes a novel legal perspective and suggests that legal sanctions could be applied. They argue that a “guest author's claim for credit of an article written by someone else constitutes legal fraud” and that, in addition, “The same fraud could support claims of ‘fraud on the court’ against a pharmaceutical company that has used ghostwritten articles in litigation.” These are potentially very serious charges that could be laid at the door of ghost and guest authors and their employers.

The second article, by medical writer Alastair Matheson [7], takes a critical look at the rules of authorship for medical journals, as laid down by the International Committee for Medical Journal Editors (ICMJE). On the basis of over 20 years' experience in the medical communications sector, he says that, in clear contrast to their intention, the authorship standards have been subverted and are being used by the pharmaceutical industry to make ghostwriting almost legitimate (note: *PLoS Medicine* is not a member of the ICMJE; we follow some of its guidelines, including those on authorship). His remedy involves fundamental revisions of the ICMJE guidelines, including the concept of origination being given comparable importance to authorship and contributorship, and that writers and companies who work on industry publications should be listed as byline authors.

Such remedies merit serious consideration, because there is no evidence that ghostwriting in the medical literature has abated; the third of the articles in *PLoS Medicine* this month [8] from someone with direct involvement in ghostwriting is unusual only because it's an example of a ghostwriter going on record. Linda Logdberg describes the nuts and bolts of how ghostwriting happens and how, as a professional medical writer in the early 2000s, she participated in it until she came across an example that clashed with her personal beliefs.

Other evidence that has come to light indicates that *PLoS Medicine* itself is not immune. First, in October 2010, Philip Davis, writing on the Society of Scholarly Publishers blog [9], asserted that an anonymous ghostwriter he had interviewed claimed to have published in many leading medical journals, including *PLoS Medicine*. (We asked Philip Davis to provide details of the papers in *PLoS Medicine*. He replied that he could not, as the ghostwriter had spoken on condition of anonymity, and to provide the details of the articles would compromise that anonymity). Second, a study by JAMA [10], reported at the Peer Review Congress in 2009 (but not yet published), that involved interviewing authors of papers published in six top medical journals, including *PLoS Medicine*, showed that 7.8% of authors from 630 articles admitted that they had lied in their authorship statements and that, in complete opposition to journal policies, had included authors who did not qualify for authorship according to guidelines (i.e., were guest authors) or had left out authors who should have been included (ghost authors). The crucial point here is that, in contrast to the documents arising from litigation such as in the PremPro case, all these accounts are recent, documenting specific examples arising in the past 5–10 years or possibly even more recently (*PLoS Medicine* is not yet 7 years old).

Such anecdotes add to the body of evidence that the medical literature continues to be systematically manipulated to promote specific products. There was perhaps one missing piece in the ghostwriting picture – that of how medical journals benefit [11]. Last year *PLoS Medicine* published an article that laid out how journals potentially benefit from ghostwritten articles. In their paper, Peter Gøtzsche and colleagues concluded that some journals derived substantial income from industry-funded trials and that industry-funded trials were in fact more likely to be cited than non-industry trials. The authors noted evidence showing that “sponsoring companies may employ various strategies to increase the awareness of their studies, including ghost authored reviews that cite them.” Journals have no more precious currency than citations.

“Medicine, as a profession, must take responsibility for this situation. Naïveté is no longer an excuse … physician-investigators should create and uphold a standard where relationships with industry are regarded as unsavory rather than sought after” [3]. This is what Adriane Fugh-Berman concluded in her article last year. To this we would add that journals too must take responsibility for their actions and start to think creatively about possible solutions, starting perhaps with those posed by Stern, Lemmens, and Matheson.

But, to be clear, the problem lies deeper than terminology. Everyone involved in the medical publishing industry, including journals, institutions, and the bodies that oversee research, need to take specific action to eradicate the seemingly endemic corrupt authorship practices that remain within the medical literature—starting by accepting the extent of the problem. Without such action, the already apparently shaky trust held by the public for the medical literature may become irrevocably damaged.
